# Evaluation of single-dose umbilical cord blood-derived mononuclear cell injection immediately and 7 days after spinal cord trauma in mice

**DOI:** 10.1016/j.clinsp.2025.100579

**Published:** 2025-01-30

**Authors:** Alex Oliveira de Araujo, Gustavo Bispo dos Santos, Raphael Martus Marcon, Maria Helena Alves Nicola, Marcela Saldanha Pereira, Fernando Barbosa Sanchez, Thiego Pedro Freitas Araujo, Alexandre Fogaça Cristante

**Affiliations:** a Department of Orthopedic Surgery, Rede SARAH de Hospitais de Reabilitação, (SMHS), Brasília, DF, Brazil; b Department of Orthopedic Surgery, Instituto de Ortopedia e Traumatologia da Universidade de São Paulo, São Paulo, SP, Brazil; c Cryopraxis Cryobiology, Espírito Santo do Pinhal, SP, Brazil; d Departament of Orthopedic Surgery, Hospital Sírio Libanês, Brasília, DF, Brazil

**Keywords:** Mononuclear stem cells, Spinal cord injury, Umbilical cord blood-derived mononuclear cell, Motor assessment, Histopathological assessment

## Abstract

**Study design:**

Experimental study utilizing a standardized Balb C mouse model.

**Objectives:**

Evaluate histological changes and motor function recovery in the acute and subacute phases of Spinal Cord Injury (SCI) in mice using human Umbilical cord blood-derived mononuclear cells.

**Methods:**

Forty mice were divided into five groups, with two receiving human Umbilical cord blood-derived mononuclear cells immediately after SCI and after 7 days, and three control groups. Motor assessment utilized BMS, MFS, and horizontal plane scales over six weeks. Necropsy evaluated macroscopic and histological spinal cord changes.

**Results:**

Histologically, Umbilical cord blood-derived mononuclear cells-treated groups exhibited significant reductions in necrosis, hemorrhage, and degeneration compared to controls. Motor recovery showed partial improvement across all groups, with no statistically significant differences in scales between intervention and control groups.

**Conclusions:**

In the acute phases of SCI, Umbilical cord blood-derived mononuclear cells applied directly to Balb C mice lesions demonstrated histological improvement but played a limited role in functional enhancement. The study highlights distinctions in the treatment's efficacy, potentially related to these cells' diverse differentiation capacities and intrinsic properties.

## Highlights

• Stem cell therapy is a promising alternative in the spinal cord injury arsenal.
• Challenges in stem cell treatment in SCI: phase uncertainty and cell type controversy.
• Umbilical cord mononuclear cells may enhance recovery via multiple mechanisms.
• Umbilical cord mononuclear cells can promote histological improvement after SCI.

## Introduction

Spinal Cord Injury (SCI) represents one of the most severe traumas in traumatology, causing significant personal losses and societal impact due to the high cost of treatment.^1^, ^2^, ^3^ The global incidence of SCI ranges from 10.4 to 83 cases per million, with the cervical spine being the most commonly affected segment. The reported prevalence oscillates between 236 and 4187 per million.^4^ Men are more prone to SCI, with occurrences typically attributable to motor vehicle collisions, falls, recreational activities, and violence.^2^

SCI can be understood as a primary injury followed by a secondary injury, resulting in a progressive cascade of tissue damage that may be exacerbated by systemic autonomic dysfunction. The suppression of this pathological process forms the basis for the pharmacological and surgical treatment of this condition.^1^^,^^2^^,^^5^

Stem cell therapy emerges as one of the promising alternatives within the therapeutic arsenal for SCI. Diverse cell types obtained from various origins hold the potential to promote tissue and functional reconstruction following SCI through a variety of mechanisms, including neuroprotection, immunoregulation, axonal regeneration, neural circuit formation, and myelin regeneration.^6^

Despite numerous experimental studies evaluating stem cell use in spinal cord injury treatment, significant challenges persist. Notably, the survival rate of transplanted cells in the spinal cord presents a significant concern. Moreover, the optimal phase of spinal cord injury for achieving the best possible outcome with stem cells remains uncertain. Additionally, there is ongoing controversy regarding the identification and selection of the most suitable cell type.^7^

The umbilical cord blood contains various types of stem cells, including hematopoietic stem cells (mononuclear), mesenchymal stem cells, and pluripotent non-hematopoietic stem cells.^8^^,^^9^ Hematopoietic cells, found in umbilical cord blood, display different differentiation stages, marked by the expression of specific antigens, like CD34.^8^ Umbilical Cord Blood-derived Mononuclear Cells (UCB-MNCs) may improve recovery through multiple mechanisms, including secretion of anti-inflammatory cytokines, release of growth factors, upregulation of matrix metalloproteinases, downregulation of tissue plasminogen activator, prevention of apoptosis and increased angiogenesis.^10^, ^11^, ^12^, ^13^, ^14^

These cells have an additional advantage in their lower immunogenicity. This is attributed to a higher proportion of immature T-cells, a reduced number of CD56+ cytotoxic T-cells, and diminished immunological memory compared to adult cells. Consequently, this leads to significantly lower rates of graft-versus-host reactions.^8^

This study aims to evaluate the histologic changes and motor functional recovery following treatment in the acute and subacute phases with UCB-MNCs in a standardized mouse model of SCI using Balb C mice.

## Methods

Forty male Balb C mice, aged between 10 and 12 weeks and weighing between 20 g to 35 g, were randomly selected for the study. All animals exhibited normal clinical conditions, as well as normal motor functions. The sample size was determined based on previous studies.^15^^,^^16^ Three animals were housed per cage, providing comfort and space for movement.

The human umbilical cord blood was ethically obtained, following informed maternal consent, from the umbilical vein of a parturient, at the Hospital das Clínicas, University of São Paulo. Mononuclear cells were separated and frozen at the Cryopraxis, Inc. laboratory (Rio de Janeiro, RJ, Brazil), where they underwent a cellular processing procedure involving cellular viability assessments, quantification, and immunophenotypic analysis. Additionally, automated procedures for microbiological testing were conducted on the processed material to detect the potential presence of aerobic and anaerobic microorganisms.

The samples, containing a pool of 1 × 10^8^ UCB-MNCs, were stored in liquid nitrogen tanks in a solution containing the cryoprotective agent Dimethyl Sulfoxide (DMSO) before being sent to the medical research laboratory, where they remained frozen until use.

The cells were rapidly thawed in a water bath at 37 °C, then diluted in a solution containing 3 mL of 20 % human albumin and 9 mL of 6 % Hemohes. Following centrifugation at 1200 rpm at 40 °C for 10 min, the pellet was resuspended in 1 mL of the solution, and cell viability was assessed using the trypan blue method (0.4 %). Following this, the final concentration was adjusted to 4 × 10^5^/40 µL.

The mice were divided into five groups, each consisting of eight animals, all subjected to experimental moderate spinal cord injury (a weight drop of 10 g from a height of 12.5 mm). The severity of the injury was determined based on the spinal cord injury standardization study in mice conducted by Borges et al. in 2018, aiming to produce a moderate injury with an acceptable complication rate.^15^ The groups were allocated as follows:

Group 1: A single injection of 4 × 105/40 µL of UCB-MNC was administered at the site of injury immediately after spinal cord contusion.

Group 2: A single injection of 4 × 105/40 µL of UCB-MNC was administered at the site of injury seven days after spinal cord contusion.

Group 3: A single injection with a volume of 40 µL of physiological saline solution was administered at the injury site immediately after spinal cord contusion.

Group 4: Animals were subjected only to spinal cord contusion.

Group 5: Control group (“sham”) involving laminectomy without spinal cord injury.

**Fig. 1:**
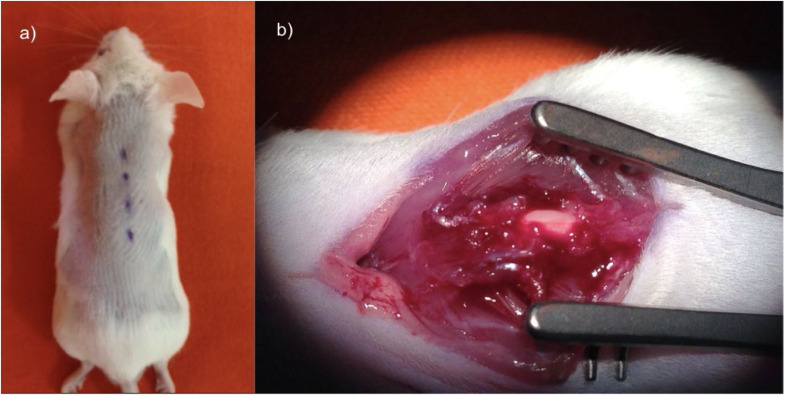
(a) Incision surgical planning. (b) T9 laminectomy with spinal cord exposure.

The animals were operated under general anesthesia.^17^ A longitudinal incision was made in the dorsal region from the T7 to T11 vertebrae. After dissection, laminectomy of the T9 vertebra was performed in a caudo-cranial direction, exposing the spinal cord (Fig. 1). The precise determination of the laminectomy level was conducted by vertebral counting. Initially, the most distal rib, corresponding to the thirteenth thoracic vertebra, was identified. Vertebral counting proceeded in the distal to proximal direction until reaching the point where the surgical intervention was performed.^15^

Throughout the surgical procedure, a surgical microscope was employed to minimize the risks of additional damage to the spinal cord, following standardized guidelines.^15^

**Fig. 2:**
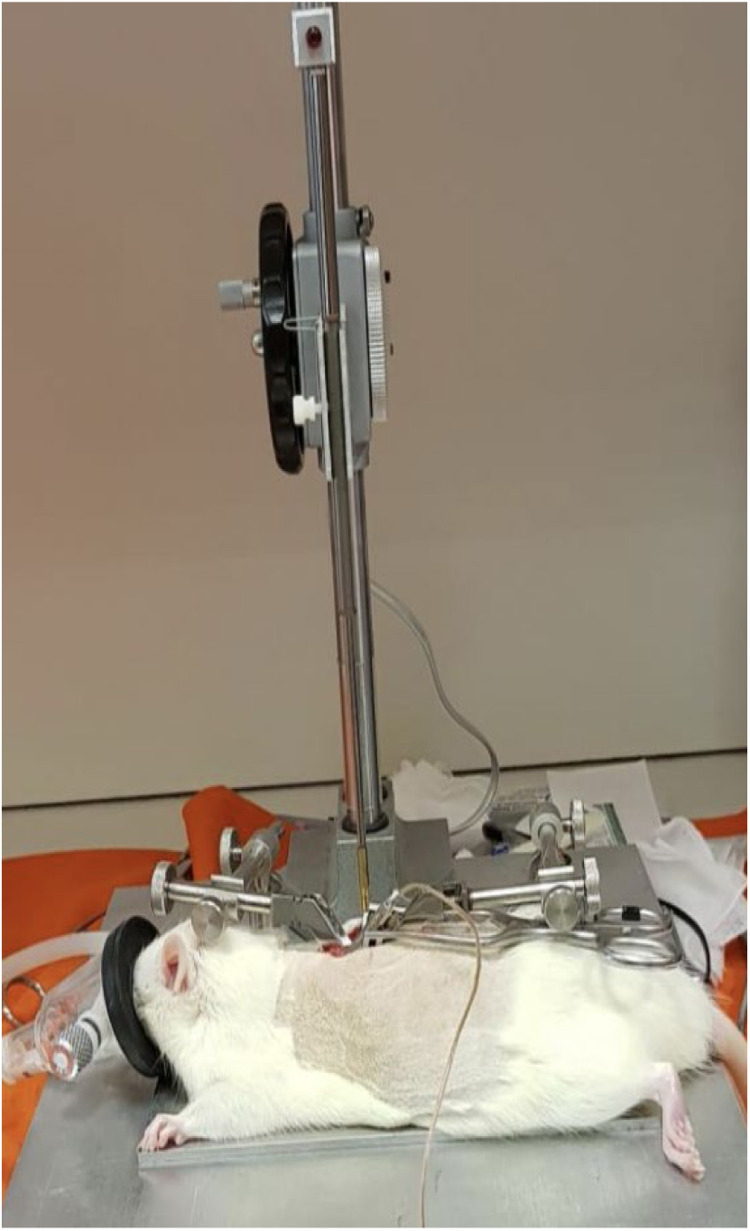
Spinal impact using the NYU Impactor.

The injuries were produced following the MASCIS (Multicenter Animal Spinal Cord Injury Study) international protocol, utilizing a New York University (NYU) Impactor (Fig. 2).^18^, ^19^, ^20^ Following the injury, either physiological saline or the stem cell solution, depending on the respective group, was applied to the lesion site.^16^

The cellular transplant was conducted in accordance with methods previously documented in the literature. Four injections of 10 μl each, administered bilaterally to the midline, both rostral and caudal to the lesion epicenter, were performed using a delicately manipulated glass micropipette, connected to a 10 μl Hamilton syringe.^21^^,^^22^

The animals were provided unrestricted access to food and water. A daily routine included bladder massage for emptying, with continuous assessment for potential complications, including monitoring for urinary infections and ensuring the well-being and health of the animals throughout the study. Additionally, analgesics and antibiotics were administered for infection prophylaxis.^15^^,^^20^^,^^23^^,^^24^

Over the 42 days of postoperative care, the animals were evaluated using three functional motor scales: the Basso Mouse Scale (BMS), the Motor Function Score (MFS), and the Horizontal Plane.^25^, ^26^, ^27^ Evaluations were visually conducted by two trained observers, blinded to each animal's study group. All mice were assessed before spinal cord injury and on days 2nd, 7th, 14th, 21th, 28th, 35th, and 42th post-injury. The lower score given by the two evaluators was chosen.

At the end of six weeks, the animals were anesthetized again and were then euthanized by cardiac perforation. The spine was removed en bloc and sent for histological analysis. The histological study involved the examination of slides stained with Hematoxylin and Eosin (HE) using optical microscopy .^15^

Three slides were prepared for each spinal cord, labeled A, B, and C. Slides A and C corresponded, respectively, to the regions proximal and distal to the injury site and were collected as individual controls for each mouse. On the other hand, fragment B corresponded to the actual injury site, differing from fragments A and C.

A pathologist evaluated the slides blindly, without knowledge of the group to which each studied animal belonged. Five parameters were assessed: necrosis, hemorrhage, hyperemia, degeneration of the white matter (cystic), and cellular infiltration. Each of these parameters was graded on a scale from 0 to 3 regarding the intensity of the alterations (0 – absent; 1 – mild; 2 – moderate; 3 – severe). Grade 1 indicated the presence of these alterations in up to one-third of the width of the spinal cord in the injury area, grade 2 indicated such alterations in one to two-thirds of the spinal cord width, and grade 3 indicated these alterations in more than two-thirds of the spinal cord width.

The evaluated parameters were detailed based on the groups and assessment time points. The authors utilized summary statistical measures, including mean, standard deviation, median, minimum, and maximum values. The comparison between parameters was performed using Generalized Estimating Equations (GEE) with a normal distribution and an identity link function. Additionally, the authors assumed a first-order autoregressive correlation matrix between assessment time points for BMS and MFS measurements. For the horizontal plane test, the number of steps was employed as a limiting factor in the data. All analyses were followed by Bonferroni multiple comparisons to identify groups or time points with significant differences.

Regarding histological parameters, the authors presented information according to groups, using absolute and relative frequencies. Group comparisons were conducted using Kruskal-Wallis tests, followed by Dunn's multiple comparisons whenever significant differences were observed.

For the analyses, the authors employed IBM-SPSS for Windows version 22.0. Data tabulation was conducted using Microsoft Excel 2013. The authors started with the null hypothesis of equality, maintaining a Type I error probability of 5 % and a Type II error probability of 20 %.

## Ethics

This study was approved by the scientific board of the Orthopedic Department of the Clinics Hospital of São Paulo University (Hospital das Clinicas da Universidade de São Paulo) and by the Animal Experimentation Ethics Committee of the School of Medicine of São Paulo College. The authors comply with the ARRIVE guidelines as well as with other guidelines established by the Canadian Council on Animal Care (CCAC), the Brazilian School of Animal Experimentation (Colégio Brasileiro de Experimentação Animal ‒ COBEA), and the National Council for Control of Animal Experimentation (Conselho Nacional de Controle de Experimentação Animal ‒ CONCEA).

## Results

During the functional analysis of mice, using both the BMS and MFS scales, no statistically significant difference was observed among the evaluated groups. The results can be observed in Table 1 and Fig. 1, Fig. 2.

**Table 1:** A comprehensive delineation of the BMS and MFS scales in accordance with distinct groups and assessment time points, accompanied by the outcomes of the comparative analyses.

Variable/Group	Time														p _Group_	p _Time_	p _Interaction_
	Pre	2 Days		Week 1		Week 2		Week 3		Week 4		Week 5		Week 6			
BMS															0.867	<0.001	0.992
Injury + Immediate Cell																	
Mean ± SD	9 ± 0	0 ± 0		0.13 ± 0.35		1.25 ± 0.71		1.38 ± 0.52		1.88 ± 0.64		2.75 ± 0.46		2.75 ± 0.46			
Median (Min; Max)	9 (9; 9)	0 (0; 0)		0 (0; 1)		1 (0; 2)		1 (1; 2)		2 (1; 3)		3 (2; 3)		3 (2; 3)			
Injury + Cell 7 days																	
Mean ± SD	9 ± 0	0 ± 0		0 ± 0		1.38 ± 0.52		1.63 ± 0.74		1.88 ± 0.64		2.75 ± 0.46		2.75 ± 0.46			
Median (Min; Max)	9 (9; 9)	0 (0; 0)		0 (0; 0)		1 (1; 2)		1.5 (1; 3)		2 (1; 3)		3 (2; 3)		3 (2; 3)			
Injury + Saline																	
Mean ± SD	9 ± 0	0 ± 0		0.13 ± 0.35		1.25 ± 0.71		1.38 ± 0.52		1.88 ± 0.64		2.75 ± 0.46		2.75 ± 0.46			
Median (Min; Max)	9 (9; 9)	0 (0; 0)		0 (0; 1)		1 (0; 2)		1 (1; 2)		2 (1; 3)		3 (2; 3)		3 (2; 3)			
Injury only																	
Mean ± SD	9 ± 0	0 ± 0		0.13 ± 0.35		1.25 ± 0.71		1.38 ± 0.52		1.88 ± 0.64		2.75 ± 0.46		2.75 ± 0.46			
Median (Min; Max)	9 (9; 9)	0 (0; 0)		0 (0; 1)		1 (0; 2)		1 (1; 2)		2 (1; 3)		3 (2; 3)		3 (2; 3)			
Sham																	
Mean ± SD	9 ± 0	9 ± 0		9 ± 0		9 ± 0		9 ± 0		9 ± 0		9 ± 0		9 ± 0			
Median (Min; Max)	9 (9; 9)	9 (9; 9)		9 (9; 9)		9 (9; 9)		9 (9; 9)		9 (9; 9)		9 (9; 9)		9 (9; 9)			
**Variable/Group**	**Time**														**p _Group_**	**p _Time_**	**p _Interaction_**
	**Pre**		**2 Days**	**Week 1**	**Week 2**		**Week 3**		**Week 4**		**Week 5**		**Week 6**				
**MFS**															**0.996**	**<0.001**	**0.995**
Injury + Immediate Cell																	
Mean ± SD	11.88 ± 0.64		0 ± 0	0.13 ± 0.35	1.5 ± 0.54		1.5 ± 0.54		1.63 ± 0.52		2.75 ± 1.39		2.88 ± 1.25				
Median (Min; Max)	12 (11; 13)		0 (0; 0)	0 (0; 1)	1.5 (1; 2)		1.5 (1; 2)		2 (1; 2)		3 (1; 4)		3 (1; 4)				
Injury + Cell 7 days																	
Mean ± SD	11.63 ± 0.74		0 ± 0	0 ± 0	1.63 ± 1.06		1.75 ± 1.04		1.63 ± 0.52		2.75 ± 1.39		2.88 ± 1.25				
Median (Min; Max)	11.5 (11; 13)		0 (0; 0)	0 (0; 0)	1 (1; 4)		1.5 (1; 4)		2 (1; 2)		3 (1; 4)		3 (1; 4)				
**Injury + Saline**																	
Mean ± SD	12 ± 0.76		0 ± 0	0.13 ± 0.35	1.5 ± 0.54		1.5 ± 0.54		1.63 ± 0.52		2.75 ± 1.39		2.88 ± 1.25				
Median (Min; Max)	12 (11; 13)		0 (0; 0)	0 (0; 1)	1.5 (1; 2)		1.5 (1; 2)		2 (1; 2)		3 (1; 4)		3 (1; 4)				
Injury only																	
Mean ± SD	12 ± 0.76		0 ± 0	0.13 ± 0.35	1.5 ± 0.54		1.5 ± 0.54		1.63 ± 0.52		2.88 ± 1.25		2.88 ± 1.25				
Median (Min; Max)	12 (11; 13)		0 (0; 0)	0 (0; 1)	1.5 (1; 2)		1.5 (1; 2)		2 (1; 2)		3 (1; 4)		3 (1; 4)				
Sham																	
Mean ± SD	12 ± 0.76		12 ± 0.76	12 ± 0.76	12 ± 0.76		12 ± 0.76		12 ± 0.76		12 ± 0.76		12 ± 0.76				
Median (Min; Max)	12 (11; 13)		12(11; 13)	12 (11; 13)	12 (11; 13)		12 (11; 13)		12 (11; 13)		12 (11; 13)		12 (11; 13)				

EEG with normal distribution and identity-link function, assuming an AR(1) correlation matrix between time points; Only the pre-moments, 2 days, 3 and 6 weeks were used for the analysis, and the Sham group was not included. SD, Standard deviation.

Table 1 demonstrates that the average behavior of the groups across the assessed time points for both scales was statistically similar (p-Interaction > 0.05). There was a statistically significant average difference between the assessment time points for both scales (p-Time < 0.05), but no statistically significant average difference was observed between the groups (p-Group > 0.05).

Both the BMS and MFS scales exhibited a mean decrease from the pre-spinal cord injury phase to the subsequent assessed time points. From day 2 onward, a statistically significant mean increase was observed at each evaluation time point, regardless of the group (*p* < 0.001).

**Fig. 3:**
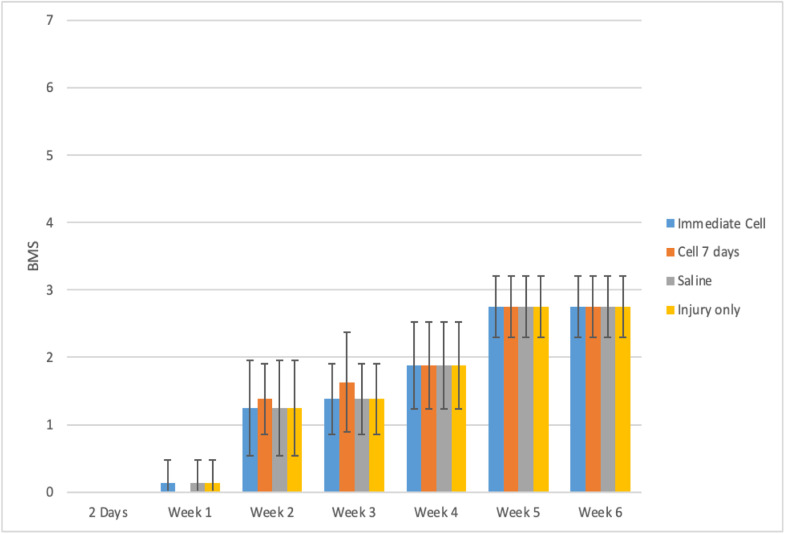
Groups behavior during the weeks according to the BMS scale.

**Fig. 4:**
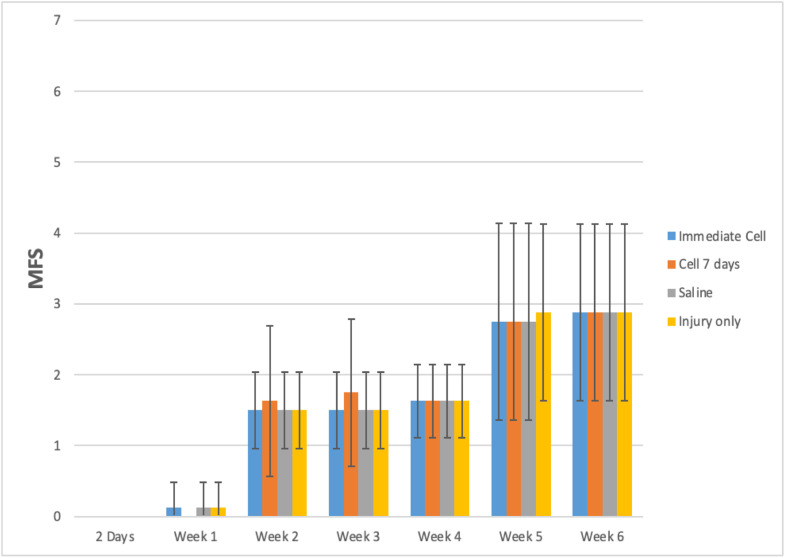
Groups behavior during the weeks according to the MFS scale.

Fig. 3, Fig. 4 illustrate a highly similar functional improvement pattern among the groups. All groups in the pre-spinal cord injury phase achieved maximum values on each scale. The Sham group maintained maximum values throughout the entire experiment. To enhance interpretability and graphical analysis, the pre-spinal cord injury time and Group 5 were excluded.

Table 2, Table 3 illustrate that all parameters of the horizontal plane exhibited statistically similar average behavior across the groups throughout the assessment time points (p-Interaction > 0.05). There was a statistically significant average difference only among the time points, irrespective of the group (p-Time < 0.001).

**Table 2:** Description of the steps and successes of the animals by group and evaluation time points, along with comparison results.

Variable	Time			p _Group_	p _Time_	p _Interaction_
	Preop	Week 3	Week 6			
**Steps**				0.698	**<0.001**	0.404
**Injury + Immediate Cell**						
Mean ± SD	46.38 ± 1.92	1.63 ± 2.26	1.63 ± 3.11			
Median (Min; Max)	47 (43; 48)	0 (0; 5)	0 (0; 8)			
**Injury + Cell 7 days**						
Mean ± SD	46.5 ± 1.69	1.63 ± 2.39	4.5 ± 3.63			
Median (Min; Max)	47 (44; 49)	0 (0; 6)	4 (0; 10)			
**Injury + Saline**						
Mean ± SD	46.75 ± 1.98	0 ± 0	3.25 ± 4.77			
Median (Min; Max)	47.5 (43; 49)	0 (0; 0)	0 (0; 12)			
**Injury only**						
Mean ± SD	46.5 ± 1.2	0 ± 0	4 ± 5.66			
Median (Min; Max)	46.5 (45; 48)	0 (0; 0)	0 (0; 12)			
**Sham**						
Mean ± SD	44.75 ± 1.49	44.75 ± 1.49	46.75 ± 1.39			
Median (Min; Max)	44.5 (43; 47)	44.5 (43; 47)	47 (45; 49)			
**Successful steps**				0.567	**<0.001**	0.158
**Injury + Immediate Cell**						
Mean ± SD	45.62 ± 1.85	1 ± 1.41	1.13 ± 2.23			
Median (Min; Max)	46 (43; 48)	0 (0; 3)	0 (0; 6)			
**Injury + Cell 7 days**						
Mean ± SD	45.38 ± 1.06	1 ± 1.41	3.38 ± 3.16			
Median (Min; Max)	45 (44; 47)	0 (0; 3)	2 (0; 8)			
**Injury + Saline**						
Mean ± SD	45.63 ± 1.51	0 ± 0	2.38 ± 3.46			
Median (Min; Max)	45.5 (43; 48)	0 (0; 0)	0 (0; 8)			
**Injury only**						
Mean ± SD	45 ± 1.07	0 ± 0	3.62 ± 5.15			
Median (Min; Max)	45 (44; 47)	0 (0; 0)	0 (0; 11)			
**Sham**						
Mean ± SD	43 ± 1.69	43 ± 1.69	46 ± 1.31			
Median (Min; Max)	43 (40; 45)	43 (40; 45)	46 (44; 48)			

EEG with normal distribution and identity-link function, assuming an AR(1) correlation matrix between time points; The same model with the number of steps as a limiting factor for Successful steps; the Sham group was not included in the analyses. SD, Standard Deviation.

**Table 3:** . Description of animal slips and errors by group and evaluation time points, along with comparison results.

Variable	Time			p _Group_	p _Time_	p _Interaction_
	Preop	Week 3	Week 6			
**Slips**				0.817	**<0.001**	0.411
**Injury + Immediate Cell**						
Mean ± SD	0.75 ± 0.71	0.25 ± 0.46	0.25 ± 0.46			
Median (Min; Max)	1 (0; 2)	0 (0; 1)	0 (0; 1)			
**Injury + Cell 7 days**						
Mean ± SD	0.88 ± 0.84	0.5 ± 0.76	0.75 ± 0.46			
Median (Min; Max)	1 (0; 2)	0 (0; 2)	1 (0; 1)			
**Injury + Saline**						
Mean ± SD	1 ± 0.76	0 ± 0	0.38 ± 0.74			
Median (Min; Max)	1 (0; 2)	0 (0; 0)	0 (0; 2)			
**Injury only**						
Mean ± SD	1.13 ± 0.64	0 ± 0	0.25 ± 0.46			
Median (Min; Max)	1 (0; 2)	0 (0; 0)	0 (0; 1)			
**Sham**						
Mean ± SD	0.88 ± 0.64	0.88 ± 0.64	0.75 ± 0.71			
Median (Min; Max)	1 (0; 2)	1 (0; 2)	1 (0; 2)			
**Errors**				0.656	**<0.001**	0.388
**Injury + Immediate Cell**						
Mean ± SD	0 ± 0	0.38 ± 0.74	0.25 ± 0.46			
Median (Min; Max)	0 (0; 0)	0 (0; 2)	0 (0; 1)			
**Injury + Cell 7 days**						
Mean ± SD	0.25 ± 0.46	0.13 ± 0.35	0.38 ± 0.52			
Median (Min; Max)	0 (0; 1)	0 (0; 1)	0 (0; 1)			
**Injury + Saline**						
Mean ± SD	0.13 ± 0.35	0 ± 0	0.5 ± 0.76			
Median (Min; Max)	0 (0; 1)	0 (0; 0)	0 (0; 2)			
**Injury only**						
Mean ± SD	0.38 ± 0.52	0 ± 0	0.13 ± 0.35			
Median (Min; Max)	0 (0; 1)	0 (0; 0)	0 (0; 1)			
**Sham**						
Mean ± SD	0.88 ± 0.64	0.88 ± 0.64	0 ± 0			
Median (Min; Max)	1 (0; 2)	1 (0; 2)	0 (0; 0)			

Translation: EEG with normal distribution and identity-link function, assuming an AR(1) correlation matrix between time points, with the number of steps as a limiting factor for slips and errors; the Sham group was not included in the analyses. SD, Standard Deviation.

The average number of steps decreased from the pre-spinal cord injury phase to the subsequent time points but increased from 3 to 6 weeks, irrespective of the group (*p* < 0.001). Slips and errors, on average, statistically increased from the pre-spinal cord injury phase to the subsequent time points in all groups but decreased from 3 to 6 weeks (*p* < 0.05). However, successful steps only increased on average from the pre-spinal cord injury phase to 3 weeks, regardless of the group (*p* < 0.001). It is important to note that the results of successful steps, slips, and errors are relative to the number of steps.

The two groups treated with stem cells exhibited a statistically significant decrease in the degrees of necrosis, hemorrhage, and degeneration when compared to the control groups.

According to Table 4, the levels of necrosis, hemorrhage, and degeneration were statistically different among the groups (*p* < 0.05), while the degree of hyperemia and infiltration did not show statistically significant differences between the groups (*p* = 0.062 and *p* = 0.090, respectively).

**Table 4:** . Description of histological parameters by group along with comparison results.

Variable	Group					p
	Immediate Cell	Cell 7 days	Injury + Saline	Injury only	Sham	
**Necrosis**						<0.001
Absent	8 (100)	8 (100)	0 (0)	0 (0)	8 (100)	
Mild	0 (0)	0 (0)	8 (100)	8 (100)	0 (0)	
**Hemorrhage**						0.001
Absent	6 (75)	6 (75)	0 (0)	1 (12.5)	6 (75)	
Mild	2 (25)	2 (25)	6 (75)	7 (87.5)	2 (25)	
Moderate	0 (0)	0 (0)	2 (25)	0 (0)	0 (0)	
**Hyperemia**						0.062
Absent	5 (62.5)	5 (62.5)	2 (25)	6 (75)	5 (62.5)	
Mild	3 (37.5)	3 (37.5)	1 (12.5)	0 (0)	3 (37.5)	
Moderate	0 (0)	0 (0)	3 (37.5)	2 (25)	0 (0)	
Intense	0 (0)	0 (0)	2 (25)	0 (0)	0 (0)	
**Degeneration**						<0.001
Absent	8 (100)	8 (100)	0 (0)	0 (0)	8 (100)	
Mild	0 (0)	0 (0)	5 (62.5)	5 (62.5)	0 (0)	
Moderate	0 (0)	0 (0)	3 (37.5)	3 (37.5)	0 (0)	
**Infiltration**						0.090
Absent	8 (100)	8 (100)	5 (62.5)	7 (87.5)	8 (100)	
Mild	0 (0)	0 (0)	1 (12.5)	0 (0)	0 (0)	
Moderate	0 (0)	0 (0)	2 (25)	1 (12.5)	0 (0)	

Kruskal-Wallis test; the Sham group (group 5) was not included in the analyses.

The degrees of necrosis, hemorrhage, and degeneration were statistically higher in the injury + physiological saline and injury-only groups compared to the injury + immediate stem cell treatment and injury + stem cell treatment after 7 days groups (*p* < 0.05).

The intervention groups displayed histological characteristics similar to the Sham group, suggesting the absence of detectable necrosis or degeneration. Additionally, there was an absence or slight occurrence of hemorrhage, in contrast to the control groups.

## Discussion

The present study followed a controlled experimental design to investigate the impacts of treatment in Balb C mice subjected to experimental moderately severe spinal cord injury. A significant difference was found in necrosis, hemorrhage, and degeneration compared to control groups, physiological saline treatment, and spinal cord injury alone (*p* < 0.05). These findings demonstrated that stem cell administration had a positive impact on the histology of the samples.

However, no significant differences were noted between the various groups in the evaluations using the BMS and MFS scales and the horizontal plane test. This lack of functional improvement suggests that while histological benefits occurred with cellular treatment, these benefits did not translate into measurable functional gains in the scales and tests used.

While this study did not demonstrate functional improvement in the intervention groups compared to controls, there are experimental studies in the literature that show functional improvement in rodents subjected to cellular treatment in acute and subacute phases.

In 2022, Shang Z. and colleagues conducted a meta-analysis of spinal cord injury studies in rats, exploring crucial factors in repair therapy. Mesenchymal stem cells from adipose tissue emerged as the most therapeutically potent cell type, with higher doses (≥ 1 × 10^6^) exhibiting significant effects. The subacute phase was identified as the optimal time for cell transplantation, and intralesional transplant emerged as the most advantageous method compared to other routes.^4^

The authors used the perilesional administration as the cell delivery route in this study. This administration route may face limitations in penetrating the Central Nervous System (CNS) due to the presence of the blood-brain barrier. While the blood-brain barrier allows the passage of essential nutrients, its permeability is restricted to many substances, including drugs that need to reach the CNS, contributing to maintaining brain and spinal cord homeostasis.^28^^,^^29^ A strategy to circumvent this limitation is the direct administration into the cerebrospinal fluid surrounding the brain or spinal cord, where diffusion in the brain is less restricted.^29^

In rodent models, the direct administration of fluids into the CNS has been performed through intracerebroventricular injections and intrathecal injections, either via the cisterna magna or lumbar region. The larger size of rats facilitates these procedures in this species, while direct administration into the CNS is generally more challenging in mice due to the increased risks of neurological injury and errors during administration owing to the smaller anatomical dimensions of structures.^29^

Peripheral stem cell transplantation has been explored, revealing some cells' ability to cross the blood-brain barrier and benefit the brain.^28^ In 2003, Saporta et al. conducted a study on Spinal Cord Injury (SCI) in rats, utilizing human umbilical cord blood mononuclear cells. The cells were administered systemically through intravenous injection either 1 or 5 days post-SCI. The group treated 5 days later exhibited a notable improvement in locomotor scores compared to both the 1-day treatment and control groups. These findings suggest that intravenous cell application during the subacute phase may offer superior benefits in reversing the clinical condition, maintaining cell migratory capacity, and contributing to neurological recovery in the injured region.^30^ However, most intravenously transplanted stem cells experience pulmonary retention after administration, resulting in limited migration to the injury site. The result is a significant reduction in the number of cells that reach the wound site, thereby limiting their effectiveness in promoting tissue healing.^4^

The transient and selective opening of the blood-brain barrier, aimed at facilitating the penetration of therapeutic biological therapies, could effectively contribute to CNS regeneration mechanisms. It has been possible to develop strategies that breach this barrier, allowing therapeutic agents to enter the central nervous system.^28^

In this study, stem cells were administered directly to the injury site during the acute and subacute phases of SCI. In these phases, there is a disruption of local microcirculation, accompanied by an intense inflammatory process that affects the blood-brain barrier.^5^ On the other hand, umbilical cord blood units were cryopreserved in a solution containing 10 % Dimethyl Sulfoxide (DMSO) and autologous plasma. DMSO is an amphipathic molecule with a highly polar domain and two apolar groups, making it soluble in both aqueous and organic environments. Penetration through most tissue membranes occurs within minutes due to a reversible alteration in protein configuration when DMSO replaces water.^31^ This agent is also used as the intracellular cryoprotectant of choice, but it may exhibit cytotoxic effects when heated.

There is a discussion in the literature regarding the ability of DMSO to increase blood-brain barrier permeability. A. Kleindienst et al. demonstrated in an experimental model of ischemic injury after middle cerebral artery occlusion in Sprague-Dawley rats that DMSO used as a solvent increased blood-brain barrier permeability, enhancing the access of drugs to the brain's extracellular space but possibly intensifying the development of vasogenic cerebral edema.^31^ The confluence of acute-phase phenomena and the presence, even in minimal quantities, of DMSO utilized in the cryopreservation of mononuclear stem cells from umbilical cord blood in this investigation could potentially enhance the permeability of the blood-brain barrier. This phenomenon may facilitate the ingress of administered cells at the injury site or neurotrophic factors produced by the cells. The improvement in the degree of necrosis, hemorrhage, and degeneration observed in the treated groups reinforces the impact that these cells had at the injury site.

In this research, the authors employed mononuclear stem cells from the umbilical cord, primarily associated with the regeneration of the hematopoietic blood system. The main objective was to investigate the potential of these cells in nervous tissue regeneration. Despite most studies involving the treatment of SCI with umbilical cord cells focusing on mesenchymal stem cells, there is evidence showing the benefits of mononuclear stem cells in SCI treatment. In a study conducted by S.I. Ryabov and colleagues in 2020, Sprague-Dawley rats with severe spinal cord injuries were treated with human umbilical cord mononuclear stem cells administered 1 day after trauma. The cells were applied both intravenously and intralesionally, both at a concentration of 2 × 10^6^. The results indicated functional and histological improvements compared to the control group.^32^

L.P. Rodrigues et al. demonstrated functional recovery in Wistar rats with spinal cord contusion treated with human umbilical cord blood mononuclear cells, either in the cisterna magna or directly at the injury site. While both groups treated 1 h or 9 days post-injury showed improved hind limb function, the acute-phase treatment group exhibited the best performance, with noticeable improvement from the second-week post-transplantation. Immunofluorescence revealed transplanted cells expressing a specific human antibody at the injury site, although no stem cell differentiation into neural or glial cells was observed.^33^

## Conclusions

This study investigated the efficacy of administering mononuclear stem cells from the umbilical cord directly onto the injury site in Balb C mice with SCI. The results indicate a more limited impact on functional improvement during the acute and subacute phases compared to other stem cell types commonly used in similar studies. Despite the observed differences, the study emphasizes the intricate interplay of factors influencing the regenerative processes in spinal cord injuries. It highlights that the therapeutic potential of stem cells extends beyond neuronal replacement, with the implanted human umbilical cord blood mononuclear cells stimulating a natural response to secondary injury and producing positive effects on histological parameters. While the functional recovery appears constrained, the study provides valuable insights into the complex mechanisms of stem cell therapy in SCI, paving the way for further investigations and refinements in treatment protocols.

## Authors’ contributions

Alex Oliveira de Araujo assumed the responsibility of designing the project, analyzing the data, composing the manuscript, and approving the final version.

Gustavo Bispo dos Santos assumed responsibility for data collection, provided support in composing materials and methods, and reviewed and approved the final version.

Raphael Martus Marcon assumed the responsibility of designing the project and meticulously reviewing and approving the final version.

Maria Helena Alves Nicola assumed responsibility for the design of the project, provided assistance in the composition of materials and methods, and reviewed and approved the final version.

Marcela Saldanha Pereira assumed responsibility for the design of the project, provided assistance in the composition of materials and methods, and reviewed and approved the final version.

Fernando Barbosa Sanchez provided support in data collection and reviewed and approved the final version.

Thiego Pedro Freitas Araujo supported data collection and reviewed and approved the final version.

Alexandre Fogaça Cristante was tasked with the design and conceptualization of the project, assisting in data analysis, as well as meticulously reviewing and approving the ultimate version.

## Declaration of competing interest

The authors declare no conflicts of interest.
